# Monitoring LFCN damage during scoliosis surgery

**DOI:** 10.1186/1748-7161-10-S1-P28

**Published:** 2015-01-19

**Authors:** Negar Behzadi Fard, Aleksandra Krajacic, Francois D Roy, Sarah Southon, Kajsa Duke

**Affiliations:** 1 Department of Mechanical Engineering, University of Alberta, Canada; 2 Alberta Health Services, University of Alberta Hospital, Canada; 3 Department of Surgery and Centre for Neuroscience, University of Alberta, Canada; 4 Division of Orthopaedic Surgery, University of Alberta, Canada

## Objective

During scoliosis surgery damage to the lateral femoral cutaneous nerve (LFCN) has been reported in 20% of patients. The purpose of this study was to characterize intraoperative pressures at the patient cushion interface and examine the LFCN somatosensory evoked potential (SSEP) to determine if there is any correlation to the incidence of LFCN injury.

## Material and methods

**Figure 1:**
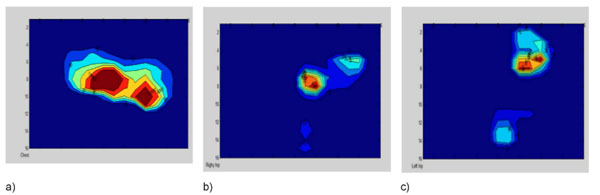
Sample pressure mat data on the a) Chest b) left hip area and c) right hip area

**Figure 2:**
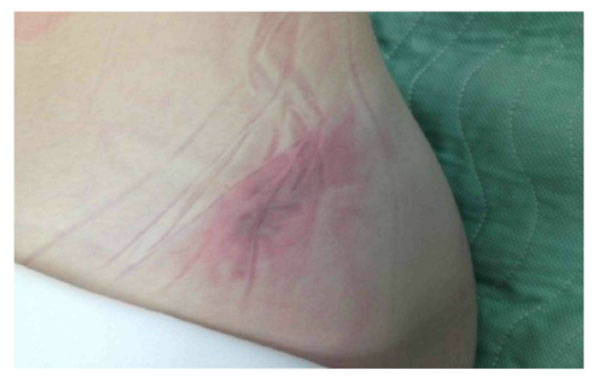
Redness over the iliac crest after surgery

Three pressure mats (FSA, Vista Medical, Winnipeg) were placed on the Jackson frame before positioning the patient. Data was continuously recorded during surgery and the average and maximum pressures on the chest, left and right hip/thigh region was calculated (Figure 1). The LFCN SSEP was tested by stimulating the anterolateral thigh and recording the evoked potentials over the somatosensory cortex. An increase in latency and/or decrease in SSEP amplitude may be indicative of LFCN dysfunction. At present, data on five patients was recorded. Post-operative evaluation of the front of the iliac crests is performed and the appearance of redness (Figure 2), blisters or pressure sores are documented. Additionally, after surgery, the patients were asked if they feel any numbness on the front of the thighs and manual tests for sensation were performed every day until discharge.

## Results

The continuous monitoring was interrupted during electrocautery. The changes in pressure on the chest pads were able to detect cyclic breathing. Increases in pressure were also noted at times such as screw placement or correction. Maximum and average pressure for the five cases is shown in Table 1. LFCN SSEP recordings were variable and the preliminary results are inconclusive. Two patients had numbness in one leg post-op. One of the injuries resolved on day four and the other was still evident on discharge.

**Table 1:** Average and maximum pressures for the five cases

	chest		Left hip		Right hip		
Case	Average (mmHg)	Max	Average (mmHg)	Max	Average (mmHg)	Max	Duration (hours)

1	51	310	23	175	22	357	6h3tf

2	42	220	29	303	34	371	6hl5'

3	68	310	29	199	27	313	4h27'

4	51	291	18	240	26	319	6hO2'

5	49	148	26	222	21	517	5h23'

## Conclusions

Pressure on the LFCN during scoliosis surgery caused numbness in 2/5 patients. Pressure mats were able to record changes during surgery. More data is required to determine if there is any correlation between elevated pressure and LFCN injury.

## Consent

Written informed consent was obtained from the patient for the image(s) used in this study. A copy of the written consent is available for review by the Editor of this journal.

